# Incidence and determinants of households’ catastrophic payments for TB care: evidence from a multi-country trial (EXIT-TB project) implemented in East Africa

**DOI:** 10.1136/bmjph-2024-001543

**Published:** 2025-02-13

**Authors:** Francis Donard Ngadaya, Doreen Philbert, Amani Wilfred, Johnson Jeremia Mshiu, Peter Binyaruka, Nicholaus P Mnyambwa, Godfather Kimaro, Amani Thomas Mori, Steve Wandinga, Blandina T Mmbaga, Bruce J Kirenga, Getnet Yimer, Sayoki Mfinanga, Esther Ngadaya

**Affiliations:** 1 National Institute for Medical Research, Muhimbili Medical Research Centre, Dar es Salaam, Tanzania, United Republic of; 2 Health System Impact Evaluation and Policy, Ifakara Health Institute, Dar es Salaam, Tanzania, United Republic of; 3 Kampala International University - Dar es Salaam College, Dar es Salaam, Tanzania, United Republic of; 4 Kenya Medical Research Institute, Nairobi, Kenya; 5 Kilimanjaro Christian Research Institute, Moshi, Tanzania, United Republic of; 6 Infectious Disease Institute, Mulago Hospital, Kampala, Uganda; 7 Center for Innovative Drug Development and Therapeutic Trials for Africa, Adis Ababa, Ethiopia

**Keywords:** economics, Public Health, Communicable Disease Control

## Abstract

**Background:**

Despite free tuberculosis (TB) services in low-resource settings which are provided under countries’ respective National TB programmes, TB patients incur substantial costs when seeking care. These costs not only act as a barrier to access but also reduce adherence to TB treatment which further affects patients’ health outcomes and poses a financial burden to households. In the context of the EXIT-TB project implementation, we nested a patient cost study aiming at estimating the costs incurred by patients when seeking TB services. In addition, we also assessed the incidence and determinants of catastrophic health expenditure (CHE) among households affected by TB.

**Methods:**

A cross-sectional analytical study was carried out in four East African Countries, namely; Tanzania, Kenya, Uganda and Ethiopia alongside EXIT-TB project implementation from 2019 to 2022. Direct and indirect costs incurred by drug-sensitive TB patients were collected after they had received TB services. Costs were considered catastrophic if they exceeded 20% of annual household income. Cost data were collected in each country’s national currency and converted to 2023 US dollars afterwards.

**Results:**

The mean total cost incurred by patients when seeking TB care were US$130.85, US$97.90, US$84.63 and US$101.60 in Tanzania, Kenya, Uganda and Ethiopia, respectively. Overall, more than half (51.81%) of the TB-affected households experienced CHE due to TB. CHE was high among TB-affected households with poor socioeconomic status. TB patients residing in Ethiopia, households with >5 members, households with TB patients as household heads, unemployed and poor socioeconomic status were among the factors associated with a high incidence of CHE (p<0.05).

**Conclusion:**

Despite the availability of free TB services in East Africa provided by the respective National TB programmes, more than half of TB-affected households experienced CHE due to TB. Our findings reinforce the need for cost mitigation policies among TB-affected households, particularly the worse offs so as to reduce the incidence of CHE and further impoverishment.

**WHAT IS ALREADY KNOWN ON THIS TOPIC**

- East African countries are embarking on healthcare reforms towards achieving universal health coverage (UHC) by 2030. One of the conditions to realise UHC is the reduction of the incidence of catastrophic health expenditure (CHE) among individuals seeking healthcare services. Given the available evidence on the cost of healthcare services, there is limited evidence on the exact incidence and determinants of catastrophic payments for tuberculosis (TB) care.

**WHAT THIS STUDY ADDS**

- The study highlights the high incidence of CHE among TB-affected households in East Africa, situating our study within the growing body of evidence highlighting the financial burden of TB, particularly among households with poor socioeconomic status (SES).
- Intensified case-finding efforts were examined, revealing their potential role in identifying TB cases earlier and highlighting the associated financial strain on households.
- Several factors were identified as being significantly associated with a higher incidence of CHE, including TB-affected households with more than five members, households with TB patients being household heads, unemployed and poor SES were among the factors associated with a high incidence of CHE (p<0.05).

**HOW THIS STUDY MIGHT AFFECT RESEARCH, PRACTICE OR POLICY**

- The rate of CHE among TB-affected households is alarmingly high. This calls for the establishment of cost mitigation policies such as economic support and socio-protection interventions so as to address financial challenges among TB-affected households, particularly the worse-offs, so as to reduce the incidence of CHE and further impoverishment.

## Introduction

 The current global End TB Strategy envision ‘a world free of TB’ with zero deaths, disease and suffering due to tuberculosis (TB) by 2035.^1^ One among the three goals is to protect TB-affected households from incurring catastrophic costs from the disease.^1 2^ This focus on ensuring that human rights, ethics and equity are protected and promoted. It also ensures the countries’ health system performance towards universal health coverage (UHC), whereby all people have access to the healthcare services they need without suffering financial hardships due to excessive out-of-pocket (OOP) payments.^3^

TB is a critical disease affecting not only the health but also the social and economic welfare of the affected households. It is one of the top 10 high-burden diseases and the 13th major cause of death globally.^4^ Before the COVID-19 pandemic, TB was the leading cause of death from a single infection, and the second cause of death after the COVID-19 pandemic ranked above HIV/AIDS.^4^ African regions account for 23% of all TB cases, making it the second WHO region with the largest number of new TB cases.^4^ The disease is highly associated with poverty, undernutrition, HIV and diabetes.^5^,^7^ Furthermore, the Global estimates show that nearly 50% of TB-affected households face costs higher than their household income.^8 9^ Such costs are deemed catastrophic when they exceed 20% of their household income.^8 9^

Among other factors, the incidence of catastrophic health expenditure (CHE) is mainly contributed by OOP payments.^10 11^ The global estimate shows 8.4% and 1.4% of households faced catastrophic expense by spending more than 10% and 25% of the total household budget for healthcare services, respectively.^10^ CHEs can result in financial distress and even impoverishment by pushing households below the poverty line.

Given the understanding of potential costs associated with the management of TB, many African governments embarked on a policy to offer free TB care and treatment services under their National TB Control Programmes. However, TB continues to impose costs to both, patients and the healthcare system as it requires routine care and long-term treatment duration with multiple health system contacts for drug collection and follow-ups.^12^ TB patients typically incur direct non-medical costs (such as transport, food and drinks) and indirect costs (productivity loss) when seeking TB services.^12^ Different studies have estimated these costs, as part of cost modelling or cost-of-illness.^13 14^ However, there is limited evidence on the effect of these costs on households’ socioeconomic status (SES). Therefore, this study aimed at estimating the cost incurred by patients when seeking TB care, the incidence and determinants of CHEs among TB-affected households in four East African countries.

## Methods

### Study design

A cross-sectional analytical study was conducted alongside the EXIT-TB project whose focus was to translate research into policy and practice through scaling-up Evidence Based Multiple Focus Integrated Intensified TB screening in the East African region.

### Study settings

The study was conducted in four East African countries, which are Tanzania, Kenya, Uganda and Ethiopia. These four countries are home to about 24.15% of the whole sub-Saharan African population. Their per capita gross domestic products are US$1076.47, US$1838.21, US$817.04 and US$936.34, for Tanzania, Kenya, Uganda and Ethiopia, respectively.^15 16^ The health systems of these countries are mainly financed through OOP payments and donor support, with small proportions of total health expenditure through government funding and health insurance schemes (as shown in table 1).^17^,^25^

**Table 1:** Selected health financing indicators in Tanzania, Kenya, Uganda and Ethiopia

	Tanzania (2019/2020)	Kenya(2019)	Uganda(2018/2019)	Ethiopia(2019/2020)
Total health expenditure per capita	US$41	US$78.6	US$33.4	US$34.50
Total health expenditure as a % of general government expenditures	22%	15.2%	7.2%	32.2%
% of insurance contribution on total health expenditure	12.1%	13.2%	4%	0.9%
% of donor/external source on total health expenditure	34.2%	17.9%	41.4%	33.9%
% of out-of-pocket household on total health expenditure	31.6%	26.1%	41.4%	30.5%
General government expenditure on health as a % of total government Spending	6.3%	6.7%	9.1%	7.8%
Total health expenditure as % of gross domestic product	4.2%	5.2%	4.3%	6.0%
Health insurance coverage	15%	17.1%	6%	28.1%

Source: National Health Accounts for Tanzania, Kenya, Uganda and Ethiopia.^17^,^25^

### EXIT-TB intervention package

The intervention package was implemented in four sub-Saharan African countries, that is, Tanzania, Kenya, Uganda and Ethiopia with the aim of complementing government efforts in increasing the number of people with active TB diagnosed. This multi-country cluster randomised trial was implemented in 28 health facilities (7 health facilities in each country). Local health facility staffs including clinicians, nurses and laboratory technicians were trained and capacitated to identify symptomatic individuals attending the health facility who are suspected to have active TB disease. In addition, the community health workers were trained to screen for TB at every facility entry points/units, such as outpatient department, the reproductive and child health department, HIV care and treatment centres, TB and diabetic clinics. Moreover, the package followed the same standard diagnostic protocol which was used under routine practice with the following additional activities; (1) TB screening, which was conducted to all individuals who initiated a visit to the health facility by using screening questionnaires, followed by chest X-ray (CXR) for individuals who presented with a history of cough of any duration, except for pregnant women, diabetic patients and advanced HIV patients who were only tested for TB using GeneXpert, (2) those with abnormal CXR were requested to submit spot sputum samples for TB diagnosis using GeneXpert and (3) all newly diagnosed TB patients were then referred and escorted to the TB clinics/direct observed therapy centre for treatment as per standard treatment guideline. Furthermore, all enrolled patients were followed up until treatment completion to ascertain their treatment outcomes and costs incurred during TB treatment.

### Study population and recruitment

A total of 1409 drug-susceptible pulmonary TB patients who were enrolled in the EXIT-TB project and responded to the patient cost questionnaire were included in this costing study.

### Inclusion and exclusion criteria

#### Inclusion criteria

All Individuals diagnosed with TB, who were enrolled in the project and sought care at the selected study sites.

#### Exclusion criteria

1. Seriously ill patients who were unable to comply with the study procedures.
2. Patients diagnosed with a multi-drug-resistant TB (MD-TB).

### Data collection and sources

Face-to-face interviews were conducted in 1409 drug-susceptible TB (DS-TB) patients enrolled under the EXIT-TB project, who had received at least 1 month of intensive phase therapy across all four countries, that is, Tanzania (n=724), Kenya (n=282), Uganda (n=104) and Ethiopia (n=299). For TB children (0–17 years), interviews were conducted with their caregivers. The questionnaire was administered by trained healthcare workers in the selected health facilities. Questions related to TB cost were validated against the WHO TB patient cost survey prior to data collection. The following patients’ information was collected: sociodemographic characteristics (age, sex, occupation and self-reported income), and overall household income. Since TB care and treatment services are provided for free, we only collected specific cost data; direct non-medical expenses (transportation, accommodation and food for the patients and their treatment supporters) and indirect costs (productivity loss incurred when seeking TB services). The indirect costs were estimated as the total period of absence from work/daily economic activities due to TB multiplied by the hourly wage of the absent worker. Data were collected using an electronic data capture (tablets) with the questionnaire translated into the country’s specific local language. Cost data were collected in each country’s national currency and converted to 2023 US dollars afterwards taking into consideration the purchasing power parity of all participating countries.

### Measurements of household living standard

This study relied on household income as a measure of household living standards. We first captured monthly income from patients and other household members, and then estimated the annual household income by multiplying the self-reported monthly income by 12 months. The proxy measures and cross-validation were conducted (eg, by comparing income with occupation) to improve the accuracy. The household income was further analysed to generate the SES in five wealth quintiles namely; poorest, second quintile, third quintile, fourth quintile and the least poor.

### Data analysis

#### CHE due to TB

To determine the incidence of household CHE due to TB, the total cost (the sum of direct non-medical costs and indirect costs) incurred by patients when seeking TB care and treatment services relative to the annual household income. Healthcare costs were deemed catastrophic if they exceeded 20% of annual household income as recommended by WHO.^26 27^

#### Factors associated with CHEs

A multivariate logistic regression analysis was performed to identify patients’ and households’ characteristics associated with the incidence of CHE due to TB. The dependent variable was binary taking a value of one for households facing CHE and zero otherwise. The selection of the potential determinants was informed by other studies exploring the correlates of catastrophic spending being conducted elsewhere.^1428^,^32^ The following patients’ level factors such as gender (male vs female), occupation (farmer, formal workers, self-employed and unemployed), age (four categories: 0–14, 15–24, 25–64 and 65+), household head (patient or non-patient); household level factors such as household size (continuous), household SES and a country of residence (Tanzania, Kenya, Uganda and Ethiopia). ORs were calculated at a 95% CI with data regarded as statistically significant only if p*<*0.05. All analyses were performed in Stata V.16 (Stata Corp, Texas, USA).

## Results

### Socioeconomic characteristics of study participants

Out of 1409 study participants from four countries, more than half (57.8%) were males, and this was consistent across countries (as shown in table 2). The majority of study participants were in the age group between 25 and 64 years with a median age (SD) of 35 years (18.06955). The sale of farm products was the major source of household income (29.59%), followed by daily wage (25.80%). The average household size was four members. Overall, the mean annual household income was US$617.15. Uganda had the highest mean household income of US$1236.48 with the lowest mean household income of US$491.59 in Kenya. Overall, more than half (52.33%) of TB-affected households were headed by TB patients. There was a regional variation in households’ socio-economic status with the majority of households falling in the poorest quintile. This reflects that most patients were from the worse-off cluster.

**Table 2:** Socioeconomic characteristics of study participants

Variable	OverallN=1409(100%)	TanzaniaN=724(51.38%)	KenyaN=282(20.01%)	UgandaN=104(7.38%)	EthiopiaN=299(21.22%)
Sex					
Male	815 (57.84)	424 (58.56)	173 (61.35)	56 (53.85)	162 (54.18)
Female	594 (42.16)	300 (41.44)	109 (38.65)	48 (46.15)	137 (45.82)
Age group (in years)					
0–14	158 (11.7)	107 (15.7)	7 (2.5)	14 (15.7)	30 (10.0)
15–24	213 (15.8)	83 (12.2)	26 (9.3)	17 (19.1)	87 (29.2)
25–64	858 (63.7)	421 (61.9)	212 (75.4)	54 (60.7)	171 (57.4)
65+	119 (8.8)	69 (10.2)	36 (12.8)	4 (4.5)	10 (3.4)
Median age (SD)	35 (18.07)				
Head of the household					
Patient	729 (52.33)	377 (52.88)	172 (61.43)	60 (58.25)	120 (40.40)
Non-patient	664 (47.67)	336 (47.12)	108 (38.57)	43 (41.75)	177 (59.60)
Main source of income					
Monthly salary	161 (11.51)	97 (13.51)	19 (6.81)	9 (8.74)	36 (12.04)
Daily wage	361 (25.80)	227 (31.62)	79 (28.32)	30 (29.13)	25 (8.36)
Business/firm earnings	190 (13.58)	99 (13.79)	44 (15.77)	13 (12.62)	34 (11.37)
Sale of farm products	414 (29.59)	143 (19.92)	57 (20.43)	36 (34.95)	178 (59.53)
Others	273 (19.52)	152 (21.17)	80 (28.67)	15 (14.56)	26 (8.70)
Mean household size	4	4	4	5	4
Mean annual household income—2023 US$ (SD)	617.15(1116.96)	654.80(1082.33)	491.59(842.24)	1236.48(2276.87)	555.57(1063.29)
Median annual household income—2023 US$ (range)	258.99(0–16 192)	245.52(0–12 276)	248.29(0–8276)	647.67(0–16 192)	280.58(0–12 518)

### Patient costs associated with TB care and treatment services

The overall mean annual cost (SD) for accessing TB services was US$114 (162.37) (table 3). Participants from Tanzania incurred the highest cost when seeking TB services (US$130.85, SD=194.38), followed by Ethiopia (US$101.60, SD=142.35), Kenya (US$97.90, SD=94.18) and Uganda (US$84.63, SD=84.63). Overall, productivity loss was the major cost driver among other components (US$43.74, SD=110.04), but there was no consistency across countries. Unlike Tanzania, the major cost driver for Kenya, Uganda and Ethiopia was transport costs, that is, US$46.60, US$53.50 and US$42.32, respectively.

**Table 3:** Patient costs associated with tuberculosis care and treatment services (in US$)

Variable	Overall	Tanzania	Kenya	Uganda	Ethiopia
Mean for annual direct non-medical costs (SD)					
Transport	38.12 (70.31)	31.78 (76.59)	46.60 (50.31)	53.50 (48.40)	42.32 (71.72)
Other costs (meals, drinks, etc)	26.90 (70.06)	28.02 (90.10)	26.27 (51.76)	33.42 (33.06)	23.58 (19.41)
Mean for annual indirect costs (SD)					
Productivity loss	43.74 (110.04)	55.12 (90.14)	31.93 (92.05)	53.48 (267.12)	29.64 (93.11)
Mean for annual total costs (SD)	114 (162.37)	130.85 (194.38)	97.90 (94.18)	84.63 (83.25)	101.60 (142.35)

### CHEs due to TB

Table 4 shows that 69.97% (678/966) of all TB-affected households experienced the incidence of CHE due to TB, meaning that they spent more than 20% of their household income in accessing TB services. The highest incidence of CHE was in Ethiopia (84.95%), followed by Uganda (78.95%), Tanzania (63.79%) and Kenya (60.35%). Across income quantiles, the incidence was high among households with poor SES.

**Table 4:** Incidence of catastrophic health expenditure due to tuberculosis by country

Variables	Overalln (%)	Tanzanian (%)	Kenyan (%)	Ugandan (%)	Ethiopian (%)
Catastrophic	678 (69.97)	259 (63.79)	137 (60.35)	45 (78.95)	237 (84.95)
Non-catastrophic	291 (30.03)	147 (36.21)	90 (39.65)	12 (21.05)	42 (15.05)
Total	966 (100.00)	406 (100.00)	227 (100.00)	57 (100.00)	279 (100.00)

### Determinants of CHEs due to TB

Table 5 shows the predictors of CHE due to TB among TB-affected households. The findings revealed that patients residing in Ethiopia (aOR=2.79; 95% CI 1.75 to 4.47), households headed by TB patients (aOR=0.65; 95% CI 0.46 to 0.91), households with more than five members (aOR=1; 95% CI 1.00 to 2.51), being unemployed (aOR=0.32; 95% CI 0.18 to 0.56), poorest (aOR=2.90; 95% CI 1.50 to 4.75) and households below an average wealth (aOR=2.54; 95% CI 1.42 to 4.55) had significantly higher odds of incurring CHEs due to TB than their counterparts. However, patients’ age had a weak association with CHEs due to TB.

**Table 5:** Determinants of catastrophic health expenditures due to TB

Variable	Catastrophic expenditure due to TB		
	aOR	(95% CI)	P value
Place of residence			
Tanzania (Ref)	1		
Kenya	0.91	(0.62 to 1.35)	0.662
Uganda	1.96	(0.93 to 4.11)	0.075*
Ethiopia	2.79	(1.75 to 4.47)	0.001**
Age groups			
0–14 (Ref)	1		
15–24	0.53	(0.26 to 1.06)	0.073*
25–64	0.61	(0.32 to 1.15)	0.126
65+	0.47	(0.21 to 1.02)	0.055*
Head of the household			
Non-patient (Ref)	1		
Patient	0.65	(0.46 to 0.91)	0.012**
Household size			
1–2 (Ref)	1		
3–4	1.23	(0.81 to 1.86)	0.331
5–6	1.59	(1.00 to 2.51)	0.047**
7+	1.78	(1.00 to 3.12)	0.046**
Occupation			
Formal workers (Ref)	1		
Farmers	0.95	(0.62 to 1.44)	0.813
Self employed	0.69	(0.46 to 1.05)	0.086*
Unemployed	0.24	(0.13 to 0.44)	0.000***
Household SES			
Least poor (Ref)	1		
Fourth quintile	1.80	(0.46 to 7.02)	0.400
Third quintile	2.54	(1.42 to 4.55)	0.002**
Second quintile	1.70	(0.10 to 2.90)	0.052**
Poorest	2.90	(1.50 to 4.75)	0.000***
Constant	1.65	(0.68 to 4.00)	0.272

Significant at ***1%, **5%, and *10% level

SES, socioeconomic status; TB, tuberculosis.

## Discussion

The study estimated total costs incurred by TB patients when seeking TB care and treatment services. In addition, we also estimated the total annual household income, the incidences of catastrophic health spending and its determinants. Overall, the mean annual cost incurred by TB patients when seeking TB care and treatment services was estimated to be US$114, with the highest costs in Tanzania, followed by Ethiopia, Kenya and Uganda. The findings are consistent with other studies that were conducted elsewhere. A study conducted by Fuady *et al* in Indonesia estimated the median (IQR) total cost incurred by households for TB-related services was US$133 (55–576) and US$2804 (1008–4325) for DS-TB and MDR-TB, respectively.^33^ Another study by Wyss *et al*^34^ in Dar es Salaam estimated the household cost for the complete treatment period to be between US$182 and US$1457. These costs are high compared with the findings of this study and this cost difference is explained by the inclusion of direct medical cost (consultation and medication) by the study which are normally incurred by the provider. Another study by Kilale *et al*^35^ investigated the economic burden of TB in Tanzania and estimated the total costs for TB care to be US$252 and US$592.5 for DS-TB and MDR-TB, respectively. This included the costs of drugs, travel, accommodation, food, nutritional supplements and productivity losses. A systematic review of 30 articles from sub-Saharan Africa that was conducted to comprehensively assess the costs incurred by TB patients when seeking TB care indicated that the total cost varied from US$1 to US$600.^6^ Moreover, total cost varied between countries, that is, Zambia (US$34 and US$68),^6^ Uganda (US$25),^6^ Ethiopia (US$34 and US$99),^36^ South Africa (US$155 and US$461),^37^ Tanzania (US$116 and US$32)^6^ and Sierra Leone (US$26).^6^

The average annual household income was much higher in Uganda (US$1236.48) compared with the rest of the countries. This was because, SES for most TB-affected households in Uganda fell between the second and the fourth wealth quintiles. In addition, Uganda had a large households size compared with other countries, that is; 60% of households had more than five household members which contributed to a high annual household income. This finding is consistent with other studies done in Uganda which suggested a monthly income of US$36 per individual per household.^29^

Overall, productivity loss was observed to be the main cost driver for TB treatment and care services whereas, for country-specific, transport costs for both patient and treatment supporters appeared to be the main cost driver for TB-related costs for Tanzania, Kenya, Uganda and Ethiopia. The costs are comparable to another study which estimated the total costs of TB care to be US$154, whereas, US$312 and US$150 were estimated for MDR-TB and DS-TB, respectively.^35^ The slight difference in TB care might be due to different types of TB (DS-TB vs MDR-TB).^35 38 39^ In addition, geographical differences can also contribute to a slight difference in costs incurred by TB patients when seeking TB services as most TB patients in the sub-Saharan African region must incur transport costs when seeking TB care. This confirms that, despite free TB care and treatment services in the region, TB-affected households are still incurring costs when accessing TB care and treatment services.

When the total cost due to TB was compared with the annual household income, we found that up to 69.97% of TB-affected households in Tanzania, Kenya, Uganda and Ethiopia faced CHE due to TB. The rate is alarmingly high and can continue to push households into impoverishment, which might further act as a barrier to access to TB services and lead to an increase in TB transmission and poor treatment outcomes among TB patients. The findings of catastrophic costs due to TB in Tanzania, Kenya, Uganda and Ethiopia are consistent with other studies conducted elsewhere.^2935 38^,^41^ In addition, the burden of CHE seems to be disproportionately concentrated among the worse-off patients (poor).

We further determined the predictors of catastrophic healthcare spending among TB-affected households. Among the selected variables, most variables appeared to influence CHE. These variables include residing in Ethiopia, patient being a household head, being unemployed and households’ SES falling in the first, third and fourth wealth quintiles. Data from previous studies have highlighted the following predictors of CHEs due to TB; hospitalisation, living in semi-urban or rural areas, having MDR-TB and the characteristics of household members were also found to be the significant determinants of CHE due to TB.^29 32 42^

### Implications for policy and practice

Despite current progress and initiatives to control TB in the sub-Saharan African region, the TB-affected households still face a higher financial burden due to TB which may act as a barrier to access and adherence to TB treatment. Therefore, countries need to develop and enforce policies that integrate socioeconomic support programmes such as subsidies or cash transfers to provide financial protection for low-income TB-affected households. Furthermore, countries should invest in and advocate for cost-cutting initiatives such as transportation subsidies for TB patients. This will reduce financial hardship due to TB, improve access to TB care and enhance treatment outcomes, aligning with the goals of UHC and the End TB Strategy.

### Strength and limitation

The study has some limitations including the use of household income as a proxy measure of households’ welfare. We acknowledge the concern about the accuracy of income data because a large proportion of the sub-Saharan population works in the informal sector, and as a result, their income tends to be highly uneven over time. We suggest further studies to use other objective measures for SES such as household consumption expenditures data. Another limitation was the use of income to estimate CHE. This is simply because income is not smooth over time, and patients are likely to hide or underestimate their true income during the interviews. We attempted to minimise this by validating their income relative to the self-reported occupation. This study only included patients with DS-TB) therefore, the estimated costs are only for DS-TB patients. In addition, the study estimated the incidence and determinants of financial catastrophe for only one disease/condition, that is, DS-TB. We suggest future studies to use aggregate OOP health spending and adjust for disease-specific thresholds to assess financial catastrophe among households so as to avoid overestimation/underestimation.

## Conclusion

We found that the majority of TB-affected households spend more than 20% of their average annual household income on TB care and treatment services across countries, which is described as catastrophic. The rate is alarmingly high and this calls for the establishment of cost mitigation policies such as economic support and socio-protection interventions among TB-affected households, particularly the worse-offs, so as to reduce the incidence of catastrophic healthcare spending and further impoverishment with an ultimate goal of improving treatment outcomes.

## Data Availability

Data may be obtained from a third party and are not publicly available.
